# Synthesis, Characterization, Thermal Properties, and Antimicrobial Activities of 5-(Diethylamino)-2-(5-nitro-1*H*-benzimidazol-2-yl)phenol and Its Transition Metal Complexes

**DOI:** 10.5402/2011/738361

**Published:** 2011-07-12

**Authors:** Vikas S. Padalkar, Vikas S. Patil, Vinod D. Gupta, Kiran R. Phatangare, Prashant G. Umape, N. Sekar

**Affiliations:** Department of Intermediate and Dyestuff Technology, Institute of Chemical Technology (Formerly UDCT), N. P. Marg, Matunga, Maharashtra, Mumbai 400 019, India

## Abstract

Synthesis and antimicrobial activities of new metal [Co(II), Cu(II), Ni(II), and Fe(II)] complexes from 5-(diethylamino)-2-(5-nitro-1*H*-benzimidazol-2-yl)phenol are described. The newly synthesized ligands were characterized by IR, ^1^H NMR, and LC-MS analysis, and metal-ligand complex formations were confirmed by using atomic absorption spectroscopy and elemental analysis. All complexes show significant in vitro antibacterial activities against *E. coli* and *S. aureus* strains and in vitro antifungal activity against *C. albicans* and *A. niger* strains by using serial dilution method. The antibacterial activities were expressed as the minimum inhibitory concentration (MIC) in *μ*g/mL. Thermal properties and electrochemical behavior of novel transition metal complexes have been studied.

## 1. Introduction

Imidazole/Benzimidazole and their derivatives are important class of organic compounds in coordination chemistry, photophysics, photochemistry, bioinorganic chemistry, and bioorganic chemistry [1–7]. 2-(2′-Hydroxyphenyl) benzimidazole (Hpbm) is an important benzimidazole-derived N, O-donor ligand. It's Zn(II), Be(II), and Al(III) derivatives are photoluminescent [8]. Hydroxyl benzimidazole shows excited-state intramolecular transfer (ESIPT) properties due to acidic protons of phenol and imidazole nitrogen (tautomerism phenomenon). It has been reported that hydroxyl benzimidazole and benzoxazole behave as a structural mimic of DNA base pair for which tautomerism may be initiated at a definite time and position within duplex DNA [9]. Structurally similar natural product bis(benzoxazole) UK-1 has been reported to posses anticancer activity, and the metal-binding studies of UK-1 indicates that benzoxazole-like compound are capable of binding a variety of biologically important metal ions [10]. Benzimidazole derivatives exhibit significant activity against several viruses such as HIV, human cytomegalovirus (HCMV) [11, 12], herpes (HSV-1) [13], and influenza [14]. Benzimidazole derivatives are unique and broad-spectrum class of antirhino/enteroviral agents such as antihistaminic [15], antipyretic [16], antiulcerative [17], antihypertensive, antiviral [18, 19], antitumor [20–24], antihistaminic [25], and antiallergic [26] and are also efficient selective neuropeptide Y Y1 receptor antagonists [27]. 

Several derivatives are reported in literature for the synthesis of benzimidazole-metal complexes for fluorescent probes and bioorganic application, but antimicrobial activities of these classes of compounds have received little attention. In the literature, there are no reports available describing synthesis and antimicrobial activities of metal complexes of 5-(diethylamino)-2-(5-nitro-1H-benzimidazol-2-yl)phenol. In this paper, we have synthesized novel ligand 5-(diethylamino)-2-(5-nitro-1H-benzimidazol-2-yl)phenol and its metal complexes and studied their antimicrobial activities.

## 2. Results and Discussion

**Scheme 1:**
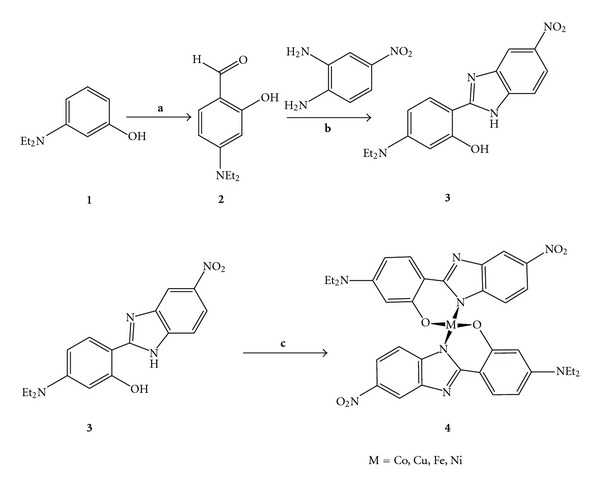
Synthesis of 5-(diethylamino)-2-(5-nitro-1*H*-benzimidazol-2-yl)phenol metal complexes. Reagent and condition:** (a)** DMF: POCl_3_, 60°C, 6h, 65% yield, **(b)** Ethanol (10 Vol.), PCl_3_ (0.01 mole), 60°C; 79% Yield, **(c)** Metal salt [Co(OAc)_2_, Cu(OAc)_2_, Ni(OAc)_2_, FeSO_4_·7H_2_O]; Methanol (5 Vol.), Triethyl amine (3-4 drop); Room temperature; 73% Yield.

The synthetic route for the preparation of 5-(diethylamino)-2-(5-nitro-1*H*-benzimidazol-2-yl)phenol metal complexes is shown in Scheme 1. *m*-(*N,N*-diethylamino)phenol (**1**) on formylation by using Vilsmeier-Haack reaction with DMF: POCl_3_ at 60°C yielded *p-N,N*-diethyl amino salicylaldehyde (**2**). The *p-N,N*-diethyl amino salicylaldehyde on further reaction with 4-nitrobenzene-1,2-diamine in ethanol and PCl_3_ at 60°C yielded the 5-(diethylamino)-2-(5-nitro-1*H*-benzimidazol-2-yl)phenol (**3**). The 5-(diethylamino)-2-(5-nitro-1*H*-benzimidazol-2-yl)phenol was reacted with different metal salt in methanol in the presence of catalytic amount of triethyl amine at room temperature to form 5-(diethylamino)-2-(5-nitro-1*H*-benzimidazol-2-yl)phenol-metal complexes. The purity of the compounds was confirmed by TLC using precoated silica gel as stationary phase, using appropriate solvent system as mobile phase and visualized under UV-light. Structures of the title compounds were confirmed by FT-IR, ^1^H-NMR analysis, and metal ligand complex formation is confirmed by atomic absorption spectroscopy. Intermediate *p-N,N*-diethyl amino salicylaldehyde formation was confirmed by its melting point. FT-IR and ^1^H NMR spectrum of 5-(diethylamino)-2-(5-nitro-1*H*-benzimidazol-2-yl)phenol showed absence of absorption band at 1670 cm^–1^ confirming the absence of aldehydic functional group by way of conversion into the corresponding benzimidazole, and absence of peak at 9.90 *δ* ppm confirmed the conversion of formyl functional group into 5-(diethylamino)-2-(5-nitro-1*H*-benzimidazol-2-yl)phenol. The FT-IR spectra of compounds **4a**–**4d** are also in complete agreement with their structure. There was sharp modification between the FT-IR spectra of the metal complexes **4a**–**4d** and the ligand 5-(diethylamino)-2-(5-nitro-1*H*-benzimidazol-2-yl)phenol (**3**), most of the bands change their pattern in the region due to coordination of the phenolic oxygen atom of OH group and nitrogen atom of imidazole ring to the metal ions. The complexation of biologically important metals with 5-(diethylamino)-2-(5-nitro-1*H*-benzimidazol-2-yl)phenol was further explored with the evaluation of their antimicrobial activity.

### 2.1. Biological Activity

All compounds were evaluated for in vitro antibacterial activities against *E. coil* and *S. aureus *strains and in vitro antifungal activity tested against *C. albicans* and *A. niger *strains by using serial dilution method.

#### 2.1.1. General

Incubator at 35 and 37°C; pipettes of various sizes (Gilson); sterile tips, 100, 200, 500, and 1000 *μ*L; sterile normal saline; sterile isosensitest agar (Southern Group Laboratory, SGL); antibiotic solutions (Sigma-Aldrich); sterile solution of 10% (v/v) DMSO in water (Sigma-Aldrich).

#### 2.1.2. Medium

Isosensitest medium was used throughout the assay, as it is pH buffered. Although NCCLS recommends the use of Mueller Hinton medium for susceptibility testing [28], the isosensitest medium had comparable results for most of the tested bacterial strains [29].

#### 2.1.3. Preparation of the Plates

Plates were prepared under aseptic conditions. A sterile 96 well plate was labelled. A volume of 100 *μ*L of test material in 10% (v/v) DMSO (usually a stock concentration of 4 mg/mL) was pipetted into the first row of the plate. To all other wells, 50 *μ*L of nutrient broth was added. Serial dilutions were performed using a multichannel pipette. Tips were discarded after use such that each well had 50 *μ*L of the test material in serially descending concentrations. To each well, 10 *μ*L of resazurin indicator solution was added. Using a pipette, 30 *μ*L of 3.3x strength isosensitised broth added to each well to ensure that the final volume was single strength of the nutrient broth. Finally, 10 *μ*L of bacterial suspension (5 × 10^6^ cfu/mL) was added to each well to achieve a concentration of 5 × 10^5^ cfu/mL. Each plate was wrapped loosely with cling film to ensure that bacteria did not become dehydrated. Each plate had a set of controls: a column with a broad-spectrum antibiotic as positive control, a column with all solutions with the exception of the test compound, and a column with all solutions with the exception of the bacterial solution, adding 10 *μ*L of nutrient broth instead. The plates were prepared in triplicate and placed in an incubator set at 37°C for 18–24 h. The colour change was then assessed visually. Any colour changes from purple to pink or colourless were recorded as positive. The lowest concentration at which colour change occurred was taken as the MIC value. The average of three values was calculated and that was the MIC for the test material and bacterial or fungal strain [30]. 

#### 2.1.4. Antimicrobial Activity

**Figure 1:**
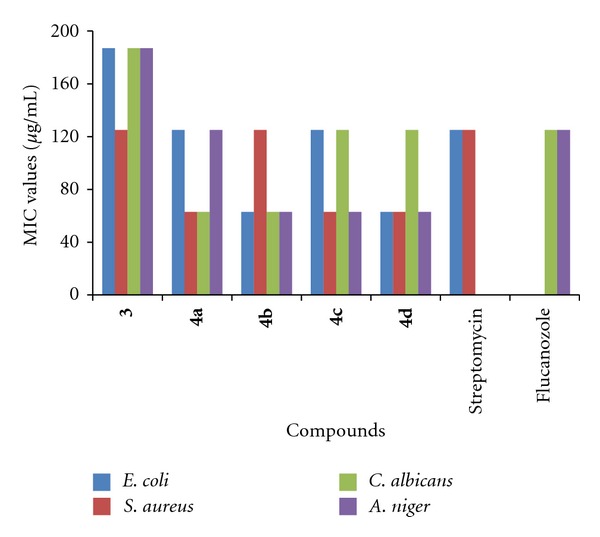
Antibacterial and antifungal activities of newly synthesized compounds indicated by MIC (*μ*g/mL) using the modified resazurin assay. MIC: Minimal inhibitory concentration values. Bacterial strain: *E. coli; S. aureus.* Fungal Strain: *C. albicans; A. niger.* Solvent used: DMSO (Dimethyl sulphoxide). Standard: Bacterial strain: Streptomycin 125 *μ*g/mL, Fungal strains: Fluconazole 125 *μ*g/mL.

The new ligand and their metal complexes were evaluated for their in vitro antibacterial activity against *E. coli* and *S. aureus *strains and in vitro antifungal activity against *C. albicans* and *A. niger *strains by using serial dilution method. The minimum inhibitory concentration (MIC) measurement determined for compounds showed significant growth inhibition zones using serial dilution method. The MIC (*μ*g/mL) values are recorded in Figure 1. The results mentioned in Figure 1 indicate that most of the tested compounds displayed variable inhibitory effects on growth of tested against bacterial strain and antifungal strain.

The metal complex **4d **showed excellent antibacterial activity against *E. coli *and* S. aureus *strains but metal-complexes **4a**–**4c** showed moderate activity against tested antibacterial strains. The inhibitory growth of metal-complexes **4a**–**4d** are almost double than novel synthesized ligand 5-(diethylamino)-2-(5-nitro-1*H*-benzimidazol-2-yl)phenol (**3**), and the compound **4b **is less active against *S. aureus *antibacterial strain. Regarding the structure-activity relationship of the novel compound 5-(diethylamino)-2-(5-nitro-1*H*-benzimidazol-2-yl)phenol complex with cobalt(II) metal showed better activity than Fe, Ni, and Cu metals. 

The results mentioned in Figure 1 showed that ligand as well as metal complexes show good inhibitory growth in case *C. albicans *as well as* A. niger *strains. All metal complexes **4a**–**4d** showed better activity than ligand (**3**). These results indicate that after coordination of biologically important transition, metal with 5-(diethylamino)-2-(5-nitro-1*H*-benzimidazol-2-yl)phenol has substantial effect on the antimicrobial activity against tested microorganism. In general, most of the tested compounds revealed better activity against the antibacterial strain (*E. coli, S. aureus*) and antifungal strain (*C. albicans, A. niger*). It was also noticed that ligand (**3**) and metal complexes **4a**–**4d** showed stronger antibacterial activity than antifungal one. 

### 2.2. Thermal Stability

**Figure 2:**
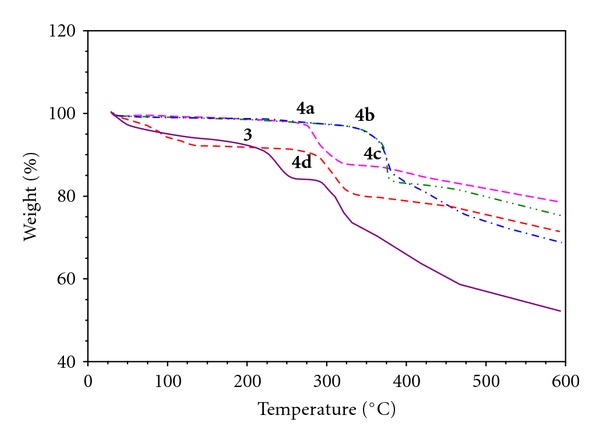
Thermo gravimetric analysis of compounds **3 **and** 4a**–**4d**.

In order to examine the thermal stability of these complexes, thermal gravimetric (TG) and differential scanning colorimeter (DSC) analysis were carried out between 40 and 600°C under a nitrogen atmosphere. The TG curves of the complexes are shown in Figure 2. The TG results indicate that the skeleton of the synthesized ligand and its transition metal complexes are stable up to 250°C. Above 250°C, the thermo gravimetric curve of the synthesized compounds show a major loss in weight. The comparisons of the *T_d_* (decomposition temperature) showed that the thermal stability of the **3** and **4a**–**4d** decreases in the order **4b **> **4c** > **4d** > **4a** > **3**. Metal complexes **4a**–**4d** is thermally more stable than ligand **3**. Metal complexes and ligand do not decompose completely even up to temperature 600°C.

**Figure 3:**
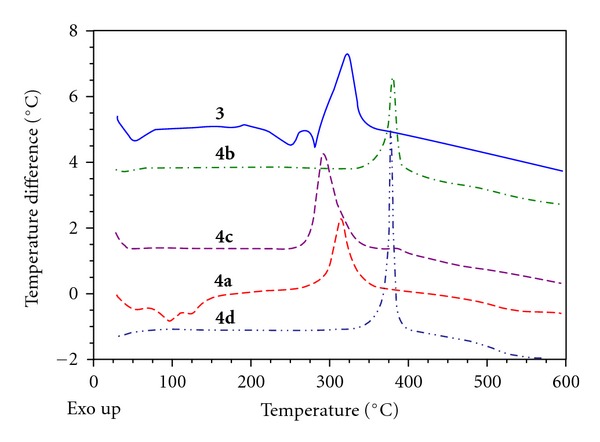
Differential scanning calorimetric analysis (DSC) overlays graph of ligand **3** and metal complexes **4a**–**4d**.

In the DSC curve, there is an endothermic peak at 240 and 270°C for ligand **3**. Metal complexes **4a**–**4d** shows exothermic peak 310, 395, 290, and 385, respectively, as shown in Figure 3.

### 2.3. Electrochemical Properties

**Figure 4:**
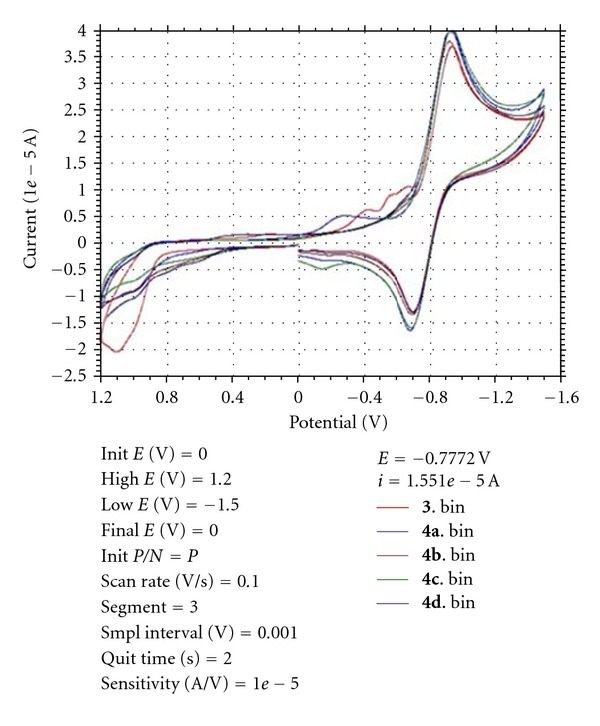
Cyclic voltammetric analysis.

The electrochemical behavior of all the complexes and ligand were studied by using cyclic voltametry (CV) in dimethyl methyl sulphoxide (0.1 M NEt_4_ClO_4_) in the potential range −1.6 to +1.2 V by using platinum auxiliary electrode and Pt disc-working electrode at ambient temperature (300 K) with no trace of decomposition as reflected in smooth curve. Cyclic voltammetric studies of the ligand **3** and complexe **4a**–**4d** in dimethyl formamide solution under nitrogen atmosphere are irreversible. The result of cyclic voltammetry of ligand closely resembles with that of metal complexes compounds, which serve as further evidences for similar structural and electronic properties Figure 4.

### 2.4. UV-Visible Properties of Ligand 3 and Metal Complexes 4a–4d

**Figure 5:**
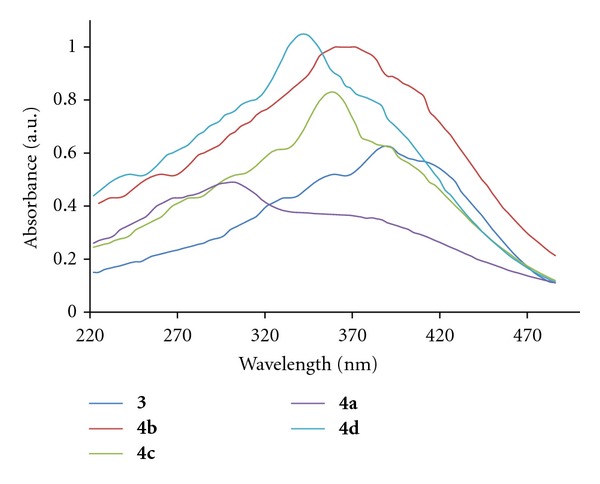
UV-Visible absorption of ligand **3** and metal complexes **4a**–**4d**.

The UV-vis absorption and emission spectra of ligand **3** and its metal complexes **4a**–**4d **were recorded in DMF at room temperature, and the compound concentrations are 1 × 10^−6^ M. The *λ*_max_ (absorbance) values of ligand **3 **is 294 (0.484), and metal complexes **4a**–**4d **were obtained as 387 (0.629), 366 (1.00), 354 (0.818), and 339 (1.043) nm, respectively. As can be seen, the absorption characteristics of metal complexes **4a**–**4d **are nearly same, while ligand absorbs in blue region as compared to complexes. The absorption spectra of ligand **3 **complexes **4a**–**4d** are shown in Figure 5.

## 3. Conclusion

In conclusion, we have synthesized new ligand 5-(diethylamino)-2-(5-nitro-1*H*-benzimidazol-2-yl)phenol and their metal complexes. These novel compounds were evaluated for in vitro antibacterial activity against *E. coli *and* S. aureus *strains as well as for antifungal activity against *C. albicans* and *A. niger* strains using serial dilution technique. 5-(diethylamino)-2-(5-nitro-1*H*-benzimidazol-2-yl)phenol metal complexes give excellent results against bacterial and fungal strain. All synthesized compounds are confirmed by FT-IR, ^1^H-NMR, and atomic absorption spectroscopy. Photophysical properties of all metal complexes are studied.

## 4. Experimental Section

### 4.1. General

All commercial reagents and solvents were procured from s.d. fine chemicals (India) and were used without purification. The reaction was monitored by TLC using on 0.25 mm E-Merck silica gel 60 F_254_ precoated plates, which were visualized with UV light. Melting points were measured on standard melting point apparatus from Sunder industrial product Mumbai and are uncorrected. The FT-IR spectra were recorded on Perkins-Elmer 257 spectrometer using KBr discs. ^1^H-NMR spectra were recorded on VXR 300-MHz instrument using TMS as an internal standard.

### 4.2. Procedure for Preparation of 4-(Diethylamino)-2-hydroxybenzaldehyde (**2**)

Phosphorous oxychloride (POCl_3_) (2.75 mL, 0.03 mole) was slowly added to dimethylformamide (DMF) (3.65 mL, 0.05 mole) at 5–10°C under the stirring. To this cooled reagent, 3-(diethyl amino)phenol (0.01 mole) was added by dissolving it into DMF (6 mL) under the stirring, and the resulting mixture was heated at 75°C for 4 hrs. The reaction mixture was cooled to room temperature and then poured into ice water (60 mL). Reaction mass was neutralised with sodium carbonate, brown colored solid separated out, filtered the separated product washed with cold water, dried, and crystallised from ethanol to get pure product (m.p. 62°C) (lit. 62–64°C).

### 4.3. Procedure for Preparation of 5-(Diethylamino)-2-(5-nitro-1H-benzimidazol-2-yl)phenol (**3**)

Phosphorus trichloride (0.33 mol) was added dropwise to a solution of the *p*-*N,N*-diethyl salicylaldehyde (0.33 mol) *N,N*-diethyl *m*-amino phenol (0.33 mol) in ethanol (50 mL), maintaining the temperature at 40–45°C. The mixture was heated at 60°C for 4 h, after the completion of reaction (monitored by TLC) cooled the reaction mass at room temperature and made alkaline to pH 8 with aqueous sodium bicarbonate solution (20% w/v). Reaction mass was concentrated under vacuum, and the solid which obtained was collected and crystallized from isopropyl alcohol.

#### 4.3.1. Spectral Analysis of 5-(Diethylamino)-2-(5-nitro-1H-benzimidazol-2-yl)phenol

IR (KBr, cm^–1^): 2991, 1620, 1520, 1338, 1149, 946, 817, 733.
^1^H-NMR (300 MHz): *δ* 1.21 (t, 6H, CH_3_), 3.42 (q, 4H, CH_2_), 6.27 (d, 1H, Aromatic H), 7.26 (d, 1H, Aromatic H), 7.29 (m, 2H, Aromatic H), 7.88 (d, 1H, Aromatic H), 7.85 (d, 1H, Aromatic H), 8.17 (s, 1H, NH), 12.43 (s, 1H, OH).
LC-MS: (327.3, 97.99%).

#### 4.3.2. FT-IR Data of Metal Complexes 4a–4d

**4a**: IR (KBr, cm^–1^): 2972, 1606, 1498, 1262, 1144, 1074, 821, 726.
**4b**: IR (KBr, cm^–1^): 2974, 1608, 1498, 1336, 1258, 1148, 1063, 1019, 818.
**4c**: IR (KBr, cm^–1^): 2973, 1607, 1497, 1336, 1258, 1149, 1019, 820.
**4c**: IR (KBr, cm^–1^): 2974, 1607, 1498, 1339, 1261, 1069, 827, 732.

### 4.4. General Procedure for Metal Complexation

To a solution of ligand, 5-(diethylamino)-2-(5-nitro-1*H*-benzimidazol-2-yl)phenol (**3**) (0.1 mole) in methanol (15 mL) was added a few drops of triethyl amine and solution of metal salts (0.05 mole) in methanol (2 mL). The reaction mixture was stirred for 24 h at room temperature. The product, thus, separated was filtered, washed with water followed by methanol, and dried to give **4a**–**4d. **Metal-ligand complexation was confirmed by using atomic absorption spectroscopy.

Atomic absorption spectra were recorded using atomic absorption spectrometer model GBC 932 (GBC Scientific Equipment, Australia). Exactly weighed dye samples were dissolved in 20 mL of dimethyl sulphoxide and diluted to 100 mL with distilled water and analyzed by GBC 932 plus atomic absorption spectrometer (AAS). Acetylene was used as fuel, and air was used as carrier gas. Certified 1000 mg/L standard solution of iron (Merck, Mumbai) was used to perform calibration using hallow cathode lamp for iron at 248.3 nm wavelength. The samples were prepared in such a manner that they will result in 2 mg/L solution containing 1 : 2 complexes. Samples were analyzed form different metals using atomic absorption spectrometer analysis.  Table 1 compares the experimental results of AAS analysis and with one calculated on the theoretical basis. The results of AAS analysis are in well agreement with the predicted results within the limitations of the experimental error, which confirms the proposed 1 : 2 metal complex stoichiometric between metal and ligand.

## Figures and Tables

**Table 1:** Atomic absorption spectrometer analysis of metal complexes.

Compounds	Molecular formula	Molecular weight	Theoretical Conc. (ppm)	Experimental Conc. (ppm)
**4a**	C_34_H_32_N_8_O_6_Co	707.60	2.00	1.94
**4b**	C_34_H_32_N_8_O_6_Cu	712.21	2.00	1.89
**4c**	C_34_H_32_N_8_O_6_Fe	704.51	2.00	1.87
**4d**	C_34_H_32_N_8_O_6_Ni	707.36	2.00	1.93
